# Research Progress of Antioxidant Nanomaterials for Acute Pancreatitis

**DOI:** 10.3390/molecules27217238

**Published:** 2022-10-25

**Authors:** Xiaoyi Zheng, Jiulong Zhao, Shige Wang, Lianghao Hu

**Affiliations:** 1 Ningxia Medical University, Postgraduate Training Base in Shanghai Gongli Hospital, Pudong New Area, No. 219 Miao Pu Road, Shanghai 200135, China; 2 Department of Gastroenterology, Changhai Hospital, Naval Medical University, No. 168 Changhai Road, Shanghai 200433, China; 3 School of Materials and Chemistry, University of Shanghai for Science and Technology, No. 516 Jungong Road, Shanghai 200093, China

**Keywords:** acute pancreatitis, oxidative stress, reactive oxygen species, antioxidant drugs, nanomaterials

## Abstract

Acute pancreatitis (AP) is a complex inflammatory disease caused by multiple etiologies, the pathogenesis of which has not been fully elucidated. Oxidative stress is important for the regulation of inflammation-related signaling pathways, the recruitment of inflammatory cells, the release of inflammatory factors, and other processes, and plays a key role in the occurrence and development of AP. In recent years, antioxidant therapy that suppresses oxidative stress by scavenging reactive oxygen species has become a research highlight of AP. However, traditional antioxidant drugs have problems such as poor drug stability and low delivery efficiency, which limit their clinical translation and applications. Nanomaterials bring a brand-new opportunity for the antioxidant treatment of AP. This review focuses on the multiple advantages of nanomaterials, including small size, good stability, high permeability, and long retention effect, which can be used not only as effective carriers of traditional antioxidant drugs but also directly as antioxidants. In this review, after first discussing the association between oxidative stress and AP, we focused on summarizing the literature related to antioxidant nanomaterials for the treatment of AP and highlighting the effects of these nanomaterials on the indicators related to oxidative stress in pathological states, aiming to provide references for follow-up research and promote clinical application.

## 1. Introduction

Acute pancreatitis (AP) is caused by the abnormal activation of pancreatic enzymes, which digest the pancreas itself and surrounding organs. It is mainly characterized by local inflammation of the pancreas and even leads to systemic organ dysfunction [1,2]. The global incidence of AP is 13 to 45 per 100,000, and the incidence is on the rise [3]. The pathogenesis of AP may be related to premature activation of trypsinogen, pathological calcium overload, mitochondrial dysfunction, pancreatic microcirculation disturbance, impaired autophagy, endoplasmic reticulum stress, and inflammatory cell infiltration [4]. However, the detailed pathogenesis of AP remains unclear. Oxidative stress plays an important role in the occurrence and development of AP, and antioxidant therapy to suppress oxidative stress by scavenging reactive oxygen species (ROS) is gaining attention from researchers [5,6,7]. However, traditional antioxidant drugs are easily oxidized and the drug delivery efficiency is low, which brings challenges to its clinical conversion application [8,9].

**Scheme 1:**
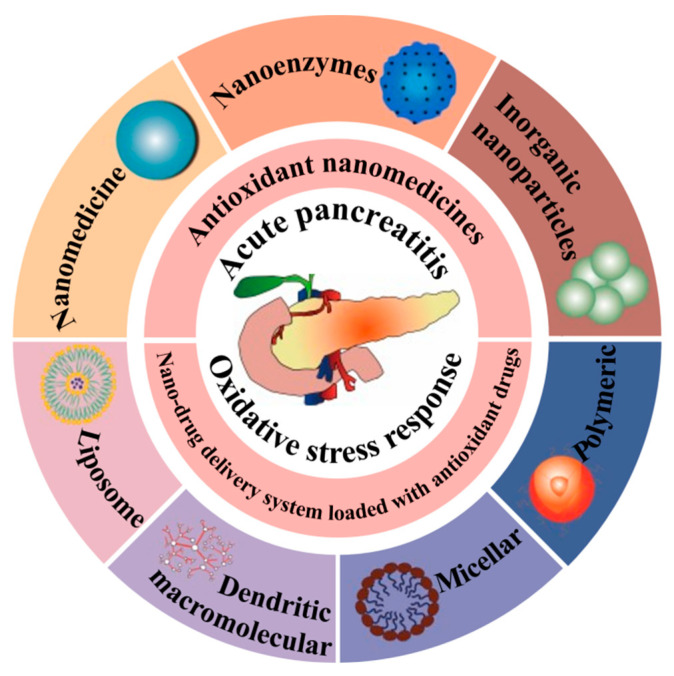
Different classes of antioxidant nanomaterials targeting oxidative stress in the treatment of acute pancreatitis.

In recent years, nanotechnology applied to the diagnosis and treatment of various diseases has become one of the most promising alternatives to conventional therapies [10,11,12,13,14]. The rapid development of nanomaterials has also provided a wide scope for the development of novel therapeutic strategies for AP. In the field of drug delivery systems, nanomaterials are generally defined in the size range of 1 to 1000 nm and have pharmacological effects or function as drug carriers [15]. The adjustment of the synthesis process can regulate the size, shape, and physicochemical properties of nanomedicines, thus conferring various advantages such as good biosafety, water solubility, and tissue permeability [16,17]. At present, a variety of nanomaterials, such as liposomes, dendrimers, biomimetic nanoparticles, and nanoenzymes, have received extensive attention in the field of biomedicine, and more and more researchers have engaged in the treatment research of AP with antioxidant nanomaterials (Scheme 1). In this review, we firstly introduce the potential pathogenesis and treatment status of AP, secondly summarize the relevant literature by searching, and introduce the application of various antioxidant nanomaterials in AP, focusing on reviewing the effects of these nanomaterials on oxidative stress-related indicators. Finally, based on the existing problems, we propose a survey on the challenges and opportunities for future nanomaterials in the treatment of AP.

## 2. The Pathogenesis and Current Treatment Status of AP

In addition to a severe inflammatory response in the pancreatic area, AP usually involves the destruction of adjacent tissues and organs. The pathogenesis of AP is a complex pathophysiological process involving multiple factors, and the specific mechanism has not been elucidated. Among the previously proposed theories, the pancreatic proteinase self-digestion theory is in the dominant position [18,19]. In recent years, the oxidative stress theory, the inflammatory mediator theory, immunogenetic theory, intestinal bacterial translocation theory, calcium overload theory, and pancreatic microcirculatory disorder theory have also received attention, among which the oxidative stress theory provides a new avenue for the comprehensive understanding of the development process of AP and its transformation to clinical treatment.

### 2.1. Oxidative Stress Response

ROS are defined as oxygen-containing intermediate metabolites with or without an unpaired electron, including oxygen radicals such as superoxide anion (O_2_^−^•), hydroxyl radical (•OH), alkoxyl radical (RO•), peroxyl radical (ROO•), nitric oxide (NO•), and non-radicals such as H_2_O_2_, hypochlorous acid (HOCl), and singlet oxygen (^1^O_2_). ROS is capable of oxidizing other components and converting them to radicals, thus causing a chain reaction leading to the production of additional free radicals [20,21,22]. ROS in the body is generated by a variety of intracellular enzymes, such as respiratory chain complex, aconitase, electron transport flavoprotein, mitochondrial glycerophosphate dehydrogenase, etc., most of which are located in mitochondria [23,24,25,26]. Other sites involved in ROS production in the body include NADPH oxidase, xanthine oxidase, peroxidase, cytochrome P450, and hemoglobin [27].

The oxidative respiratory chain of mitochondria is regarded as the main producer of ROS [25]. To regulate the intracellular ROS balance, the organism has emerged with various antioxidant strategies. Therefore, the body’s enzymatic antioxidant system and non-enzymatic antioxidant system can work together to maintain the balance of oxidation and antioxidant system. The enzymatic antioxidant system includes catalase, superoxide dismutase, glutathione peroxidase, etc., and the non-enzymatic antioxidant system includes glutathione, vitamin C, vitamin E, selenium, etc. [28]. In a pathological state, an imbalance between the oxidation and antioxidant systems occurs, resulting in the massive production and accumulation of ROS, which is called oxidative stress injury [29].

### 2.2. Oxidative Stress and AP

During the development of AP, oxidative stress involves regulating a variety of key cellular processes that play an important role in the progression of pancreatic damage. In the calcium overload theory, when AP occurs, the structure and function of the cell membrane are damaged, and the extracellular Ca^2+^ can flow into the cell through the opened Ca^2+^ channel, causing intracellular Ca^2+^ overload. Such Ca^2+^ overloading further inhibits the mitochondrial function, which paralyzes energy-dependent Ca^2+^ pumps, premature activates the digestive enzymes, and triggers a severe inflammatory response [30]. It can be seen that the Ca^2+^ overload of pancreatic acinar cells is a key link in promoting the development of AP, and the participation of ROS aggravates this process. The reasons for Ca^2+^ overload caused by ROS are: (1) these highly activated substances react with cell membrane phospholipids and trigger the lipid peroxidation chain reaction on the cell membrane, which finally leads to the disintegration of the cell membrane and destroys its barrier function. Moreover, the Ca^2+^ overload can further increase the cell membrane permeability and attenuate its fluidity, which in turn leads to an influx of Ca^2+^ and cell death [31,32]. (2) ROS disrupts the balance of the cellular regulation of Ca^2+^ entry, release, and exit processes, which subsequently affects mitochondrial function and activates various digestive enzymes. For example, IP3R and Ryanodine receptors both contain multiple ROS-sensitive cysteine residues [33,34,35]. (3) ROS affects the activity of Ca^2+^ ATPase, the enzyme that can reduce the intracellular calcium ion concentration by oxidizing the thiol-rich active center of the enzyme [36,37]. In addition, the ROS can also activate phospholipase A_2_ and accelerate the hydrolysis of membrane phospholipids, which also reduces the activity of Ca^2+^ ATPase [38,39]. In the inflammatory factor theory, various inflammatory mediators or cytokines are responsible for the spread of pancreatitis inflammation and the dysfunction of surrounding multiple organs. A large number of released inflammatory factors and chemokines trigger the cascade effect of inflammation through trigger-like action and finally lead to the transformation of AP from local lesions to systemic inflammatory response syndrome and multiple organ failure [40]. Moreover, ROS can activate nuclear factor-κB (NF-κB), c-Jun N-terminal kinase, mitogen-activated protein kinase (MAPK), and other inflammatory signaling pathways, resulting in increased release of TNF-α, IL-1, IL-6, and other inflammatory factors and chemokines and accelerating the progress of inflammation [5,41,42]. According to the pancreatic microcirculation disorder theory, in the early stage of AP, total pancreatic blood flow often decreases, capillaries atrophy, endothelial cell permeability increases, and hemodynamic changes lead to circulatory disorders such as blood viscosity and micro thrombosis [43]. ROS can further damage the integrity of vascular endothelial cells and increase the permeability of capillaries by promoting the massive secretion of inflammatory factors and the adhesion, activation, and migration of inflammatory cells, and finally aggravate the obstacles of pancreatic microcirculation [4]. In addition, ROS can further initiate cell necrosis and apoptosis through various pathways. For example, H_2_O_2_ can recruit the translocation of Bax/Bak proteins to mediate the release of cytochrome c, the activation of caspase-3, and DNA fragmentation [44,45,46].

### 2.3. Antioxidant Drugs for AP

A growing number of studies have shown that oxidative stress inhibition by scavenging ROS with antioxidant drugs can effectively mitigate the progression of AP [47,48,49,50]. For example, N-acetyl-l-cysteine (NAC) is a thiol compound that participates in the regulation of cellular redox reactions by providing sulfhydryl groups. In the rat AP model, NAC can downregulate chemokines, monocyte chemotactic protein 1, macrophage inflammatory protein 2, and other expressions, thereby effectively inhibiting oxidative stress-induced pancreatic damage [51,52,53]. As another paradigm, melatonin has been widely used in the treatment of experimental AP rats. Melatonin possesses a powerful free radical scavenging activity and antioxidant activity that can alter the activity of enzymes such as superoxide dismutase (SOD) and glutathione peroxidase (GPx) [54,55]. In another study, Lewinski et al. found that resveratrol, an efficient free radical scavenger, can effectively prevent damage to pancreatic alveoli in AP rats by reducing ROS production and inflammatory cell infiltration [49]. In addition, ascorbic acid, α-tocopherol, β-carotene, selenium, butylated hydroxyanisole, and other antioxidant drugs can adjust the balance between oxidants and antioxidants, and also have certain effects on the treatment of AP [8,56]. Although the above-mentioned antioxidant drugs can alleviate or prevent the development of AP, they have limitations such as poor drug stability, low bioavailability, short half-life, limited targeting capacity, and dosage-related side effects. Therefore, how to improve the stability of antioxidant drugs, enhance their ROS scavenging efficiency and reduce the dosage of drugs have become hot spots in recent years for AP treatments.

## 3. Advantages of Nanomaterials in the Treatment of AP

Nanomaterials offer a variety of advantages over traditional pharmaceutical formulations. Due to the increased vascular permeability at the inflammation site, nanomaterials can penetrate through the vascular endothelial gap to the lesion site and selectively target the pancreatic inflammatory tissue by crossing the blood-pancreatic barrier, cellular biofilm barrier, and other body barriers [57,58]. Moreover, nanomaterials offer unique advantages in terms of bioavailability, the release of slow and controlled release drugs, and uptake by target cells or tissues due to their small particle size, large specific surface area, good solubility, and the ability to be absorbed by cells in the form of cellular drinks [17,59].

Moreover, through chemical or physical modifications, nano-drugs can take advantage of changes in the inflammatory microenvironment (e.g., pH, ROS, and trypsin) to achieve targeted controlled release favorable to drug absorption [60,61,62]. Notably, drug carriers can increase the effective blood concentration time and in vivo safety, which helps to reduce the frequency of drug administration and reduce the toxic side effects of drugs. This is because the nano-scale carriers can increase the hydrodynamic radius, reduce the glomerular filtration rate, and prolong the half-life of the drug [63,64].

## 4. Antioxidant Nanomaterials for AP

### 4.1. Nano-Drug Delivery Systems Loaded with Antioxidant Drugs

#### 4.1.1. Liposome Drug Delivery Systems

**Figure 1:**
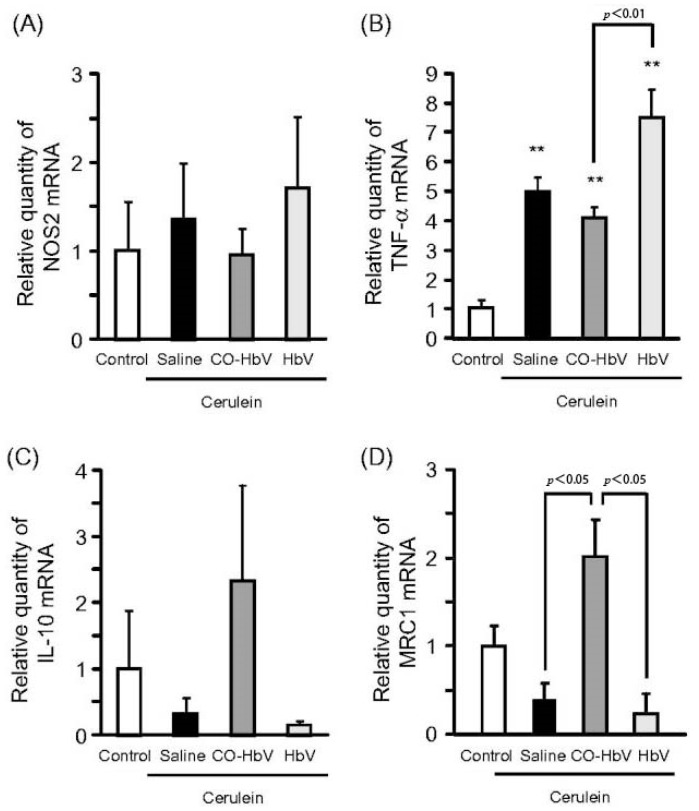
Effect of CO-HbV on M1 and M2 type macrophage polarization in the pancreas in a model of acute pancreatitis. (**A**) mRNA expression levels of NOS2 (M1 macrophage marker) in the physiological saline group, HbV group and CO-HbV treatment group. (**B**) mRNA expression levels of TNF-α (M1 macrophage marker) in the physiological saline group, HbV group, and CO-HbV treatment group. (**C**) mRNA expression levels of IL-10 (M2 macrophage marker) in the physiological saline group, HbV group, and CO-HbV treatment group. (**D**) mRNA expression levels of MRC1 (M2 macrophage marker) in the physiological saline group, HbV group, and CO-HbV treatment group. (The expression levels of the above mRNAs were measured in pancreatic tissues collected at the 12th hour after the start of mouse modeling with cerulein). ** *p* < 0.01 versus control. Reprinted from [70]. Copyright 2018.

Antioxidant drug delivery systems are capable of loading antioxidant drugs through physical or chemical modifications and improving the distribution, regulating the drug release, and weakening drug toxicities of drugs at the site of pancreatic inflammation [4,58]. Liposomes are a widely used nano-drug delivery system. Typical liposomes have a bilayer structure similar to a cell membrane and are closed spheres consisting of a hydrophilic polar head group and a hydrophobic non-polar tail group [65]. Liposomes have diameters ranging from 20 nm to 10 μm, which can be modified to encapsulate lipid-soluble drugs, water-soluble drugs, and amphoteric drugs [66]. It has been shown that carbon monoxide (CO) can inhibit oxidative stress and inflammatory responses by scavenging ROS at sites of inflammation. However, the bioavailability of gaseous CO was restricted by its inhomogeneous distribution in the organism [67,68]. Since hemoglobin (Hb) can efficiently be conjugated with CO, Maruyama et al. prepared a nano-vesicle drug carrier (CO-HbV) by wrapping CO-bound Hb with liposomes, which increased the biocompatibility and half-life of Hb, as well as the CO delivery efficiency [69]. The results of the AP mouse model showed that CO-HbV nanodrugs have good biosafety and, as a novel CO donor, it delays the AP progression and systemic organ damage by removing ROS and reducing neutrophil infiltration at inflammatory sites. Further study showed that CO-HbV can modulate the CO release at the inflammation site and target the macrophage polarization to promote its conversion to the M2 type, which leads to the increased expression of anti-inflammatory cytokines and decreased expression of pro-inflammatory cytokines [70] (Figure 1).

#### 4.1.2. Dendritic Macromolecular Drug Delivery Systems

**Figure 2:**
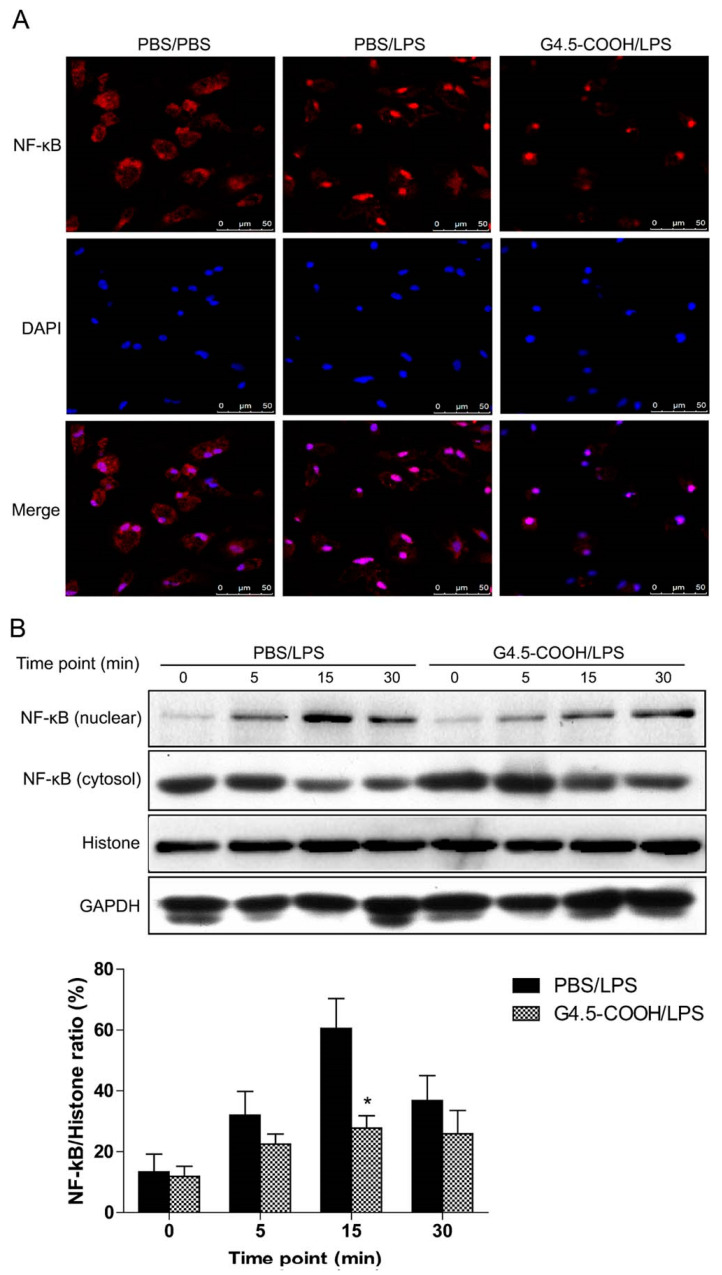
Inhibition of pancreatic inflammatory response by G4.5-COOH is associated with inhibition of NF-κB nuclear translocation in macrophages. (**A**) Macrophages were preincubated with PBS or G4.5-COOH, followed by lipopolysaccharide (LPS) for 30 min after stimulation of inflammation for confocal assays. Alexa Fluor-labeled NF-κB protein is shown in red, and DAPI-labeled cell nuclei are shown in blue. After LPS stimulation for 0, 5, 15, and 30 min, cytoplasmic and nuclear proteins were extracted from the macrophages, and the expression of NF-κB protein. (**B**) The expression of NF-κB in the cytoplasm and nucleus of macrophages was detected by Western blot and the results were quantified. * *p* < 0.05, compared with PBS pretreated group. Reprinted (adapted) from [78]. Copyright 2015, with permission from the American Chemical Society.

Polyamidoamine (PAMAM) dendrimers consisting of diamines (e.g., ethylenediamine) and branched surface groups have been used as drug carriers for gene drugs or insoluble molecules through various functional modifications [71,72]. Jiang et al. reported that synthetic PAMAM-glutathione (GSH) was prepared by encapsulating the antioxidant drug GSH. Owing to the good transmembrane ability and efficient drug-loading rate of PAMAM dendrimers, PAMAM-GSH can effectively reduce intracellular ROS levels [73]. In addition, several studies have found that dendrimers have various biological activities and functions such as activation of monocytes, inhibition of cyclooxygenase expression, and reduction of nitric oxide production [74,75,76,77]. To explore the potential of dendrimers in AP therapy, Tang et al. investigated the protective effect of PAMAM dendrimers with two different surface groups, Generation 4.5 anionic PAMAM dendrimers (G4.5-COOH) and Generation 5 neutral PAMAM dendrimers (G5-OH), on pancreatic injury in a mouse model of cerulein-induced AP [78]. The results showed that both G4.5-COOH and G5-OH dendrimers significantly reduced the infiltration of macrophages in pancreatic tissue and attenuated the inflammatory response in pancreatic tissue. Moreover, the plasma leukocyte count and monocyte count were significantly reduced in the G4.5-COOH group compared with the G5-OH treated mice, suggesting that the former may have a better in vivo protective effect in AP. The mechanism may be related to the G4.5-COOH group’s involvement in inhibiting the NF-κB signaling pathway in macrophages (Figure 2).

#### 4.1.3. Micellar Drug Delivery Systems

**Figure 3:**
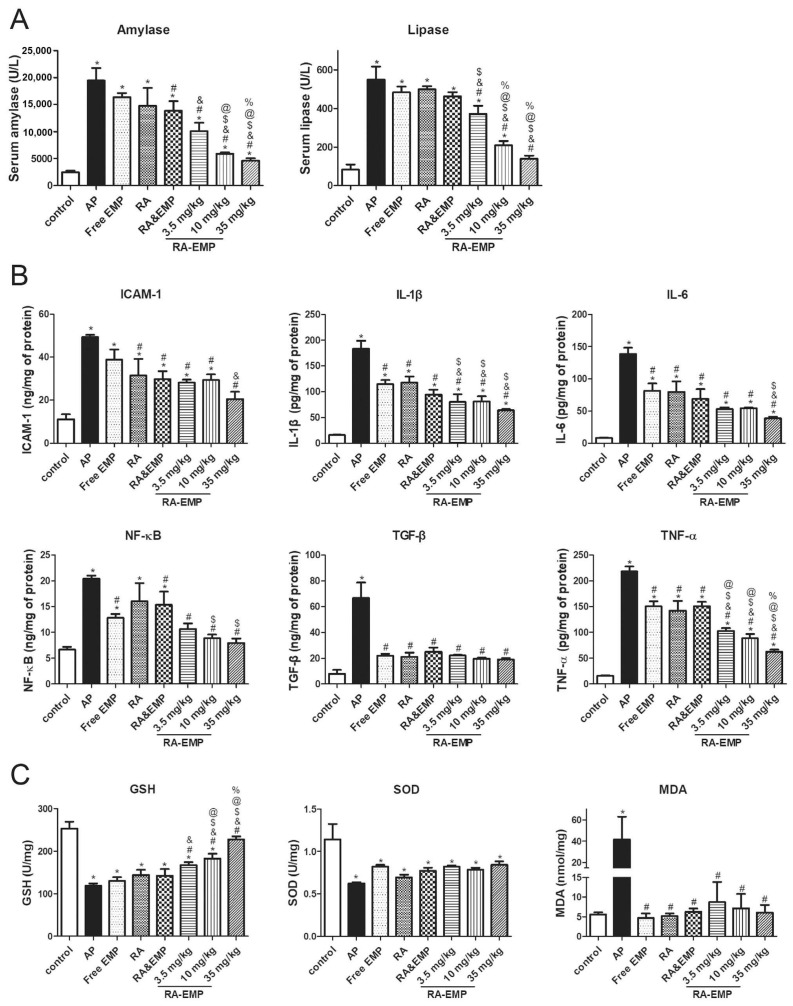
Biochemical assay, proinflammatory cytokines assay, and tissue oxidative stress analysis. (**A**) Amylase and lipase levels in serum after treatment with different concentrations of micelles (in order: physical mixture group; 3.5 mg/kg group; 10 mg/kg group; 35 mg/kg group). (**B**) Levels of pro-inflammatory cytokines in pancreatic tissues of experimental groups treated with different concentrations of micelles (in order: physical mixture group; 3.5 mg/kg group; 10 mg/kg group; 35 mg/kg group). (**C**) GSH, SOD, and MDA contents in the pancreas. * *p* < 0.05 vs. healthy control group; # *p* < 0.05 vs. AP control group; & *p* < 0.05 vs. free EMP group; $ *p* < 0.05 vs. RA group; @ *p* < 0.05 vs. RA&EMP group; % *p* < 0.05 vs. RA-EMP 3.5 mg/kg group. Reprinted from [83]. Copyright 2022, with permission from Elsevier.

The micelles formed by self-assembly of amphiphilic surfactants or polymers can encapsulate water-insoluble drugs to form nano-sized colloidal dispersions, usually between 5 and 100 nm in size. The micelles synthesized from natural compounds also have the advantages of good biocompatibility and in vivo degradability [79]. Studies have shown that Empagliflozin (EMP), a clinically used oral hypoglycemic agent, plays an important role in the treatment of type 2 diabetes. Notably, EMP has potential value in the treatment of AP because it also has good antioxidant and anti-inflammatory effects; however, the poor water solubility of EMP affects its bioavailability [80,81,82]. Li et al. found that Rebaudioside A (RA), an extract from *Stevia rebaudiana*, has an amphiphilic molecular structure and can instantaneously self-assemble into ultra-small micelles in an aqueous solution [83]. Therefore, using RA as a carrier, they prepared a novel EMP self-assembled nano-micellarized formulation (RA-EMP). The RA-EMP has the characteristics of simple preparation, good storage stability, high solubility, and high EMP encapsulation efficiency. In vivo, experimental studies demonstrated that RA-EMP significantly increased the oral bioavailability of EMP, and the expression of serum GSH was significantly enhanced. Moreover, their results showed that the expression of inflammatory factors such as serum IL-6, IL-1β, and TGF-β was significantly decreased, and the inflammation of pancreatic tissue was significantly reduced in the RA-EMP group (Figure 3).

#### 4.1.4. Polymeric Drug Delivery Systems

Polymer nanoparticles, including synthetic polymers, such as poly lactic-co-glycolic acid (PLGA), polyethyleneimine (PEI), polyethylene glycol (PEG), etc., as well as natural polymers, such as chitosan (CTS), silk fibroin (SF), etc., are commonly used nano-drug carriers. These polymers were compatible with most drugs and their degradation products have good biocompatibility [84,85]. Studies have confirmed that curcumin (CUR), as a natural polyphenol derivative, can effectively scavenge ROS and has potential applications in the treatment of oxidative stress-related diseases. However, the application of CUR was restricted by its limited bioavailability and poor stability [86,87]. Using an improved solvent evaporation method, Anchi et al. developed PLGA-based CUR-loaded particles (CuMPs) [87]. In vitro drug release and in vivo pharmacokinetic studies confirmed the superior efficacy of CuMPs over repeated oral or intraperitoneal administration of CUR, which may be related to the sustained release of CUR from CuMPs. Further, the levels of GSH and Nrf-2 in the CuMPs treatment group of the cerulein-induced AP mice were significantly higher than in the control, and the levels of inflammatory factors such as IL-1β were decreased, suggesting the effective attenuation of the oxidative and nitrosative stress and inflammatory responses of pancreatic inflammatory sites.

**Figure 4:**
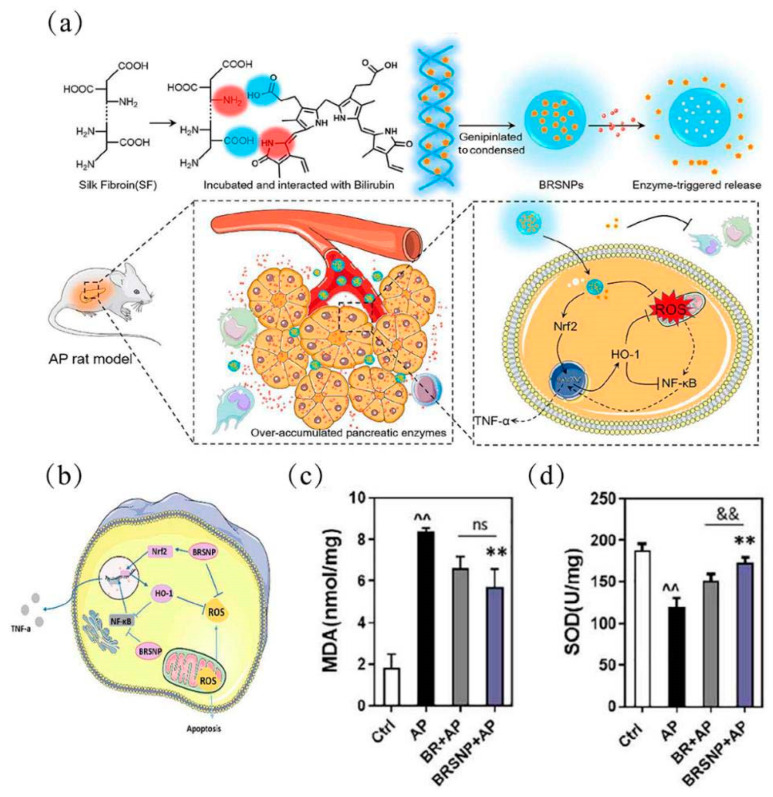
(**a**) Schematic diagram of bilirubin-loaded silk fibroin nanoparticles (BRSNPs) for the treatment of experimental AP. (**b**) Schematic representation of the antioxidant and anti-inflammatory mechanisms of BRSNPs in pancreatic alveolar cells. (**c**) MDA levels in rats after treatment of AP in the BRSNPs group and all other groups. (**d**) SOD levels in rats after treatment of AP in the BRSNPs group and all other groups. && *p* < 0.01 compared with BR group. ** *p* < 0.01 compared with model group. ^^ *p* < 0.01 compared with normal control rats. Reprinted from [88]. Copyright 2020, with permission from Elsevier.

Similarly, bilirubin is of great interest as an endogenous antioxidant compound. Low levels of bilirubin in tissues can adequately scavenge ROS and reduce intracellular oxidative stress levels; however, the use of bilirubin is still limited by its low water solubility and hyperbilirubinemia-related toxicity [88,89,90]. Therefore, Yao et al. designed bilirubin nanoparticles (BRSNPs) loaded with SF by the co-precipitation method [88]. In the inflammatory microenvironment of the pancreas, BRSNPs can be degraded by a variety of protein hydrolases, resulting in an enzyme-responsive rapid bilirubin release. In addition, BRSNPs not only improved the water solubility of bilirubin but also avoided jaundice caused by large amounts of free bilirubin. Rat experiments showed that the BRSNPs inherited the antioxidant and anti-inflammatory effects, which reduced the in vivo Malondialdehyde (MDA) levels and increased the SOD levels of rats. Moreover, BRSNPs inhibited the development of oxidative stress and inflammation by modulating NF-κB and Nrf2/HO-1 pathways, and this stimulus-responsive nanoparticle targeting the pancreatic inflammatory microenvironment provided a novel drug delivery option for the treatment of AP (Figure 4).

In recent years, although nanoparticles have been widely studied and applied in the field of treating inflammatory-like diseases, the disadvantages exhibited by different nanoparticles such as cytotoxicity, immunogenicity, and poor targeting have limited the wide application of nanoparticles [91,92,93]. In contrast, nanoparticles obtained by special integrated modification of nanomaterials using natural cell membranes, such as red blood cell membranes and immune cell membranes, can effectively reduce the cytotoxicity and immunogenicity of nanoparticles while improving the histocompatibility and biological targeting of nanoparticles, a significant advantage that has received widespread attention from researchers [94,95,96].

Celastrol (*Celastrus orbiculatus*, CLT) has a variety of anti-inflammatory, antioxidant, and anti-cancer activities and has been widely studied in the treatment of many diseases [97,98,99]. To explore the value of CLT in AP, Zhou et al. developed a neutrophil membrane-wrapped PEG-PLGA/CLT nanoparticles (NNPs/CLT) [100]. Since neutrophils can spontaneously target the inflammation site, NNPs/CLT overcome the blood–pancreatic barrier of pancreatic inflammation and exert their anti-inflammatory and anti-oxidative stress effects to effectively alleviate the disease progression. In another study, Hassanzadeh et al. confirmed that ferulic acid (FA) can effectively scavenge ROS, and to solve the problem of poor bioavailability and solubility of FA, it was encapsulated in neutrophil membrane-wrapped SF-based nanoparticles (FA-SF-NPs) [101]. The as-prepared FA-SF-NPs can selectively deliver FA to the pancreatic lesion site and increase the in vivo SOD, GPx, and reduced glutathione/glutathione disulfide bond levels, suggesting the potential therapeutic value of FA-SF-NPs for AP. Table 1 shows the research progress of nano-drug delivery systems loaded with different antioxidant drugs.

### 4.2. Antioxidant Nanomedicines

#### 4.2.1. Nanomedicine Particles

In addition to loading or encapsulating antioxidant drugs with various nanocarriers, the drugs with antioxidant properties can be assembled into nanoparticles under external mechanical strength. Directly engineering drugs into nanoparticles is not only simple and efficient, but also can improve the disadvantages of poor solubility and bioavailability of the drugs and control the continuous release of the drugs to guarantee their therapeutic functions. For example, Abizaid et al. reported that cinnamic acid (CA) and its phenolic derivatives, such as caffeic acid and erucic acid have good antioxidant and anti-inflammatory activities [102]. Using a simple grinding method, they prepared the cinnamic acid nanoparticles (CA-NPs). This method can improve the bioavailability of cinnamic acid. In addition, CA-NPs can downregulate redox-sensitive signaling pathways such as NLRP3, NF-κB, and ASK1/MAPK, which further protect the pancreatic alveolar cells from the destruction of pancreatic inflammation.

#### 4.2.2. Nanozymes

Natural enzymes have the characteristics of diverse catalytic activity and substrate, However, natural enzymes still have some shortcomings such as high costs, poor thermal stability, low recycling rate, etc. [103,104,105]. Compared with natural enzymes, the nanozymes (which are nanomaterial-based artificial enzymes) have the characteristics of a simple preparation process, good stability, and high recycling efficiency [106]. At present, a variety of nanomaterials with unique enzymatic catalytic activities have been reported, such as polypyrrole nanoparticles, Au nanoparticles, Fe_3_O_4_ nanoparticles, carbon nanotubes, etc. [107,108,109,110]. The discovery of these nanozymes provides a new research field for the treatment of AP. Zheng et al. prepared Prussian blue nanoenzyme (PBzyme) using a polyvinylpyrrolidone modification method [111]. The PBzyme showed good dispersion stability and biocompatibility under physiological conditions, which effectively scavenged ROS in the pancreatic acinar cell line AR42J cells level. Further in vivo attempts on a Caerulein-induced mouse AP model confirmed that the PBzyme decrease the MDA levels and increases the SOD and GSH levels. The AP therapeutic outcome may be related to the inhibition of the TLRs/NF-κB signaling pathway.

**Figure 5:**
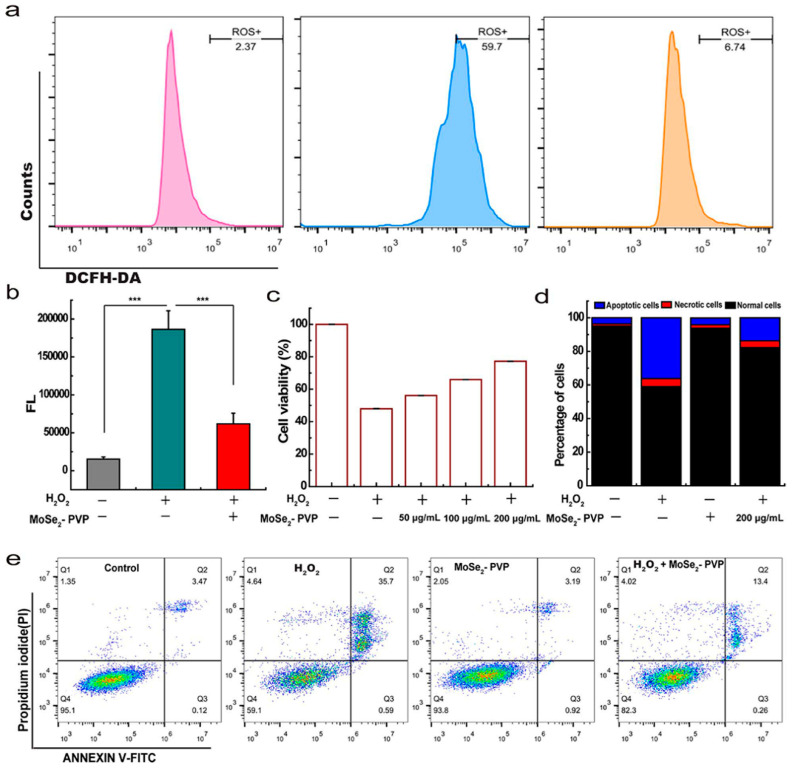
(**a**) Quantitative analysis of ROS levels in RAW264.7 cells (left: healthy cells; middle: H_2_O_2_-treated RAW264.7 cells; right: H_2_O_2_/MoSe_2_-PVP NPs-treated 264.7 cells). (**b**) Quantitative analysis of ROS levels in RAW264.7 cells (H_2_O_2_ −: without the H_2_O_2_; H_2_O_2_ +: with the H_2_O_2_; MoS_2_-PVP −: without the MoS_2_-PVP). (**c**) Histogram of RAW264.7 cells vitality after treatment with 500 μM H_2_O_2_ and different concentrations of MoSe_2_-PVP NPs. (**d**) Histogram of apoptosis and necrosis in untreated and MoSe_2_-PVP NPs-treated RAW264.7 cells (**e**) of cell apoptosis and necrosis distribution in untreated and MoSe_2_-PVP NPs-treated RAW264.7 cells. *** *p* < 0.001, Representative graphics are shown, Reprinted from [115]. Copyright 2022.

Moreover, nanozymes can mimic the activities of a variety of enzymes, and their advantages are gradually attracting research attention. Some studies have reported that certain nanoenzymes have similar properties to peroxidase (POD), catalase (CAT), GPx, and SOD. By scavenging the in vivo ROS in the body and maintaining intracellular redox homeostasis, they can not only achieve the effect of alleviating various types of inflammation but also reduce the burden of antioxidant enzymes under pathological conditions [112,113,114]. In normal organisms, the antioxidant enzyme system requires the participation of multiple enzymes to maintain redox homeostasis in cells. To simulate this multi-enzyme complex system, we prepared polyvinylpyrrolidone-modified molybdenum selenide nanoparticles (MoSe_2_-PVP NPs) by a hydrothermal one-pot method for the treatment of AP [115]. MoSe_2_-PVP NPs were prepared simply and efficiently, which exhibited good colloidal stability, biocompatibility, and biodegradability, and mimicked the activities of native CAT, SOD, POD, and GPx. Our results showed that MoSe_2_-PVP NPs exhibited excellent antioxidant properties to protect cells from ROS damage in vitro, and the in vitro experiments also confirmed the significant therapeutic effect of these MoSe_2_-PVP NPs on AP in mice (Figure 5).

Recently, two-dimensional transition metal nanosheets have shown good potential for anti-inflammatory and antioxidant applications due to their ultra-thin structure and high specific surface area. Wang et al. prepared a two-dimensional polyvinylpyrrolidone (PVP)-modified selenomolybdenum nanosheet (MoSe_2_@PVP NSs) by a simple one-pot method [116]. As an artificial nano-antioxidant, MoSe_2_@PVP NSs were able to mimic the activities of CAT, SOD, POD, and GPX over a wide temperature range, and scavenge ROS, and RNS with high efficiency and heat resistance. In an animal model of AP, MoSe_2_@PVP NSs can downregulate inflammation-related factors such as IL-6, IL-1β, and TNF-α, confirming the potential ability of two-dimensional nanosheets in the treatment of AP.

With the progress of research, the advantage that inorganic nanoparticles can not only mimic multi-enzyme activity, but also participate in antioxidant reactions in vivo by changing their elemental valence has also received the attention of scholars, which is beneficial to achieve a better therapeutic effect on experimental AP. For example, Khurana et al. noted that a lanthanide rare earth element, yttrium (Y), can interchange between multiple valence states and promote free electron migration to reduce ROS production [117]. Moreover, its counterpart yttrium oxide (Y_2_O_3_) nanoparticles (NYs) can mimic the activity of CAT and SOD, thus exhibiting strong antioxidant properties. In addition, NYs can inhibit inflammatory cell recruitment and modulate the Nrf_2_/NF-κB pathway to restore mitochondrial and endoplasmic reticulum homeostasis and delay the disease progression. In another study, Khurana et al. found that cerium (Ce) can switch between Ce^3+^ and Ce^4+^ oxidation states [118]. Therefore, the prepared cerium oxide (CeO_2_) nanoparticles (NC), which can also mimic the CAT and SOD activities, exhibited strong ROS scavenging properties. Animal experiments further confirmed that NC improved AP in mice by reducing MDA levels and increasing GSH levels in vivo.

**Figure 6:**
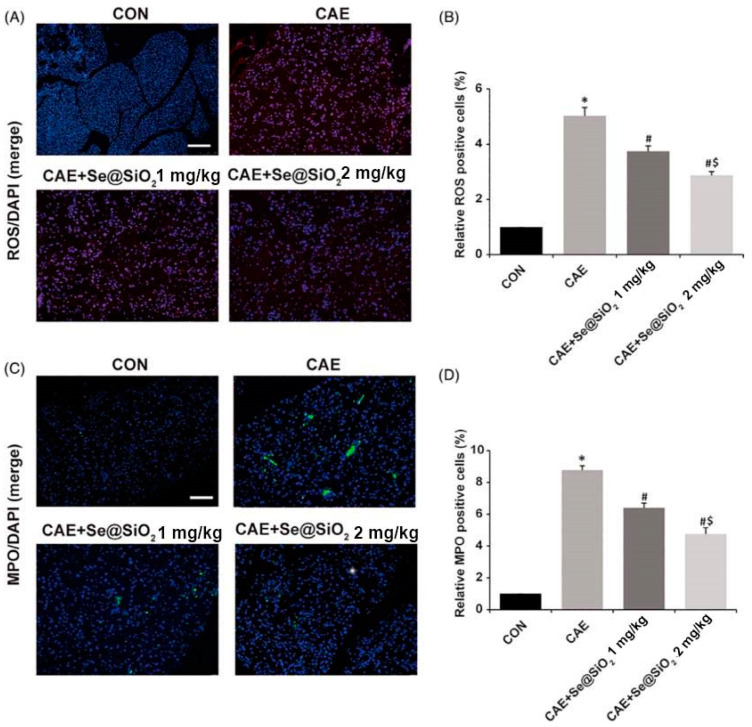
Se@SiO_2_ nanoparticles can reduce pancreatic oxidative stress in mice with cerulein-induced AP. (**A**) Immunofluorescence staining of ROS produced in pancreatic tissue (red: ROS; blue: nucleus). (**B**) The relative number of ROS-positive cells in each group. (**C**) Myeloperoxidase (MPO) staining results of pancreatic samples from each treatment group (green: MPO; blue: nuclei). (**D**) The relative number of MPO-positive cells in each group. *p* < 0.05 vs * the CON group, # the CAE group, and $ the CAE + 1 mg/kg Se@SiO_2_ group. Reprinted from [120]. Copyright 2021, with permission from John Wiley & Sons, Inc.

Selenium (Se) is a potent antioxidant that can be involved in the enzymatic antioxidant system in vivo to remove harmful ROS and protect cells. Hakeem et al. developed Se nanoparticles (nano-Se) with antioxidant activity [119]. The experiment showed that nano-Se not only increased the serum Se and GSH levels and pancreatic Se content but also effectively reduced MDA and minimized AP-induced pancreatic damage. In another study, Fan et al. developed porous silica (SiO_2_)-coated ultra-small selenium nanospheres (Se@SiO_2_ nanospheres). The results indicated that the levels of ROS, Myeloperoxidase, (MPO), and MDA in the experimental group were significantly reduced after treatment with Se@SiO_2_ nanospheres (Figure 6). Moreover, the levels of GSH and SOD increased, which effectively alleviated the damage to the pancreas caused by oxidative stress. In addition, Se@SiO_2_ nanospheres can also target TLR4/Myd88/p-p65 and NQO1/Nrf2/HO-1 pathways to reduce inflammatory damage to the pancreas [120]. Table 2 shows the effects and mechanisms of different types of nanomaterials in the treatment of AP.

## 5. Summary and Outlook

In summary, antioxidant drug treatment strategies for AP have received increasing attention; however, conventional drugs are still limited by disadvantages such as poor bioavailability and have not yet been used in clinical practice on a large scale. With the continuous development of nanotechnology, various novel and multifunctional nanomaterials have been applied in the clinical research of AP. Antioxidant nanomaterial is a combination of nanomaterials and drugs, which provides new avenues for the treatment of AP. We also noted that conventional nanomaterials are generally produced by physical or chemical means, but there are some disadvantages of using these two methods, including the need for expensive equipment, harmful chemicals, and control of reaction conditions. Recently, biological approaches to obtain nanomaterials have gained wide recognition and significant interest based on the production of biological nanomaterial derived from natural organisms, microorganisms, microalgae, enzymes, and plant extracts, among others. This approach can provide a safe, non-toxic, energy-efficient, environmentally friendly, and low-cost synthesis method with unique advantages in terms of stability and biocompatibility of organisms. Therefore, future research could focus on the role of bio-nanomaterials in the development of AP through their anti-oxidative stress and anti-inflammatory effects. Although a variety of nanomaterials have been developed for the treatment of AP, most of them have only been validated in simple animal models, and large-scale, multicenter studies have not yet been conducted. To this end, future research can focus on developing more optimized nanomedicine carriers and antioxidant nanomedicines to maximize their advantages in AP therapy, and address the challenges, such as reducing in vivo toxic effects, improving drug targeting, and increasing biodegradability of nanomaterials in AP therapy. Moreover, mechanistic and statistical studies should also be emphasized to bridge the research gap and promote the clinical translational application of antioxidant nanomaterials.

## Figures and Tables

**Table 1:** Nano-drug delivery system loaded with different antioxidant drugs.

Nanocarrier	Drug	Characteristics	Ref.
liposomes	Carbon monoxide (CO)	A by-product of inducible heme oxygenase-1, which effectively regulates inflammation and oxidative stress	[69]
liposomes	Carbon monoxide (CO)	It has powerful cytoprotective effects and multiple biological functions, such as antioxidant and anti-apoptotic effects, while inhibiting the expression of pro-inflammatory cytokines and increasing the production of anti-inflammatory cytokines	[70]
Dendrimers	G4.5-COOH; G5-OH	Multiple biological activities, high drug loading rate, high biocompatibility, low cytotoxicity and hemolysis, low protein interactions	[78]
Micellar	Empagliflozin (EMP)	Anti-oxidative stress and anti-inflammatory effects involve modulation associated with high mobility group box 1 signaling	[83]
Polymeric (PLGA)	Curcumin (CUR)	A polyphenol derivative from the traditional Indian plant turmeric (Curcuma longa), Cur enhances NF-κB pathway, c-Jun N-terminal ki-nases, and other related signaling pathways exert their anti-inflammatory, anti-oxidative stress	[87]
Polymeric (SF)	Bilirubin	Endogenous antioxidant compounds derived from the catabolism of heme, and low levels of bilirubin in tissues can adequately scavenge ROS and reduce intracellular oxidative stress	[88]
Polymeric (PEG-PLGA)	Celastrol (CLT)	A compound isolated from the roots of the thunder god vine with a variety of activities including NF-κB inhibition, anti-inflammatory, antioxidant, and anti-cancer	[100]
Polymeric (SF)	Ferulic acid (FA)	A multifunctional bioflavonoid that is more effective in scavenging free radicals, stimulating cytoprotective enzymes or the immune system	[101]

**Table 2:** Different types of nanomaterials used for the treatment of acute pancreatitis.

Types and Sizes of Nanomaterials	Animal Model	Effects and Cellular Mechanisms	Ref.
liposomes (CO-HbV) (280 nm)	CDE (choline-deficient ethionine supplemented); mice	Reduction of TNF-α and IL-1β, neutrophil infiltration, MPO, and NO_2_-Tyr (anti-oxidative stress; anti-inflammatory)	[69]
liposomes (CO-HbV) (280 nm)	Caerulein; mice	Transformation of macrophage phenotype, reduction of HMGB1, MPO, and NO2-Tyr levels, and reduction of neutrophil infiltration (anti-oxidative stress; anti-inflammatory)	[70]
Dendrimers (G4.5-COOH; G5-OH) (5 nm)	Caerulein; mice	Inhibits nuclear translocation of NF-κB and reduces the expression of inflammatory factors such as IL-1β and IL-6 and infiltration of macrophages (anti-inflammatory)	[78]
Micellar (RA-EMP) (4.703 ± 0.114 nm)	L-Arginine; mice	Decrease the expression of GSH, SOD, IL-6, IL-1β, and TGF-β in serum and increase the expression of MDA (anti-oxidative stress; anti-inflammatory)	[83]
Polymeric (CuMPs) (14.34 ± 5.98 µm)	Caerulein; mice	Increases GSH and Nrf-2, decreases serum amylase and lipase and levels of inflammatory factors such as IL-1β (anti-oxidative stress; anti- nitrosative stress; anti-inflammatory)	[87]
Polymeric (BRSNPs) (268 ± 6 nm)	L-Arginine; rat	Inhibition of NF-κB pathway, activation of Nrf2/HO-1 pathway, reduction of pro-inflammatory cytokines, and inflammatory cell infiltration (anti-oxidative stress; anti-inflammatory)	[88]
Polymeric (PEG-PLGA/CLT) (150 nm)	Injection of 3% sodium taurine into the pancreatic duct after aseptic dissection; rat	Reduces serum amylase and MPO levels inhibit the NF-κB pathway and reduce expression of inflammatory cytokines (anti-oxidative stress; anti-inflammatory)	[100]
Polymeric (FA-SF-NPs) (186 nm)	Biliopancreatic duct ligation; rats	Decreased expression levels of SOD, GPx, GSSH/GSSG, IL-β, and TNF-a and increased expression levels of MDA (anti-oxidative stress; anti-inflammatory)	[101]
Particles (CA-NPs) (50–90 nm)	L-Arginine and gamma radiation; rats	Downregulation of NLRP3, NF-κB, and ASK1/MAPK signaling pathways; decreased caspase-3 expression levels (anti-oxidative stress; anti-inflammatory)	[102]
Nanoenzymes (PBzyme) (60 nm)	Caerulein; mice	Reduced MDA levels, increased SOD and GSH levels, and inhibited TLRs/NF-κB signaling pathway (anti-oxidative stress; anti-inflammatory)	[111]
Nanoenzymes (MoSe_2_-PVP NPs) (119.39 ± 13.94 nm)	Caerulein; mice	Mimics the activity of CAT, SOD, POD, and GPx eliminates multiple ROS, reduces serum amylase levels and inflammatory factors (anti-oxidative stress; anti-inflammatory)	[115]
Nanoenzymes (MoSe_2_@PVP NSs) (86.278 ± 11.82 nm)	Caerulein; mice	Mimics the activity of CAT, SOD, POD, and GPx eliminates ROS and RNS, reduces serum amylase levels, IL-6, IL-1β, and TGF-β levels (anti-oxidative stress; anti-inflammatory)	[116]
Inorganic (NYs) (159.2 ± 7.5 nm)	Caerulein; mice	Mimics CAT and SOD activity, inhibits inflammatory cell infiltration, regulates Nrf2/NF-κB pathway, and restores mitochondrial and endoplasmic reticulum homeostasis (anti-oxidative stress; anti-inflammatory)	[117]
Inorganic (NC) (82 ± 5.4 nm)	Caerulein; mice	mimicking CAT and SOD activities, decreasing the levels of p65-NF-κB, Hsp27, and Hsp70, and upregulating the expression of Nrf2, SOD1, and NQO1 (anti-oxidative stress; anti-inflammatory)	[118]
Inorganic (nano-Se) (20–60 nm)	L-Arginine; rat	Inhibition of NF-kB pathway and reduction of serum MDA, IL-1β, IL-6, and TNF-a expression levels (anti-oxidative stress; anti-inflammatory)	[119]
Inorganic (Se@SiO_2_) (50 nm)	Caerulein; mice	Targeting TLR4/Myd88/p-p65 and NQO1/Nrf2/HO-1 pathways to reduce ROS, MPO, and MDA levels and increase GSH and SOD levels (anti-oxidative stress; anti-inflammatory)	[120]

## Data Availability

Not available.
